# Chlorogenic acid regulates the expression of protein phosphatase 2A subunit B in the cerebral cortex of a rat stroke model and glutamate-exposed neurons

**DOI:** 10.1186/s42826-024-00196-5

**Published:** 2024-03-01

**Authors:** Ju-Bin Kang, Hyun-Kyoung Son, Dong-Ju Park, Yeung-Bae Jin, Phil-Ok Koh

**Affiliations:** Department of Anatomy and Histology, College of Veterinary Medicine, Research Institute of Life Science, Gyeongsang National University, 501 Jinjudaero, Jinju, 52828 South Korea

**Keywords:** Cerebral ischemia, Chlorogenic acid, Middle cerebral artery occlusion, PP2A subunit B

## Abstract

**Background:**

Ischemic stroke is a serious neurological disorder caused by blockages in cerebral artery. Protein phosphatase 2A (PP2A) is a phosphatase that performs a critical role in cell signaling and growth. PP2A subunit B acts as a neuroprotective agent in the nerve system. Chlorogenic acid, which is mainly found in roasted coffee, has antioxidant, anti-inflammatory, and anti-apoptotic effects. We hypothesized that chlorogenic acid modulates PP2A subunit B expression in ischemic stroke models and glutamate-mediated neurons. Middle artery occlusion (MCAO) surgery was operated and chlorogenic acid (30 mg/kg) or phosphate buffer saline was treated 2 h after MCAO. The cerebral cortex was collected 24 h after surgery and the change of PP2A subunit B expression was analyzed. Glutamate and/or chlorogenic acid were treated in cultured neurons, further study was performed.

**Results:**

A decrease in PP2A subunit B expression in MCAO animals was identified. Chlorogenic acid alleviated this decrease due to ischemic injury. Moreover, the number of PP2A subunit B-positive cells in the ischemic cerebral cortex was significantly decreased, chlorogenic acid alleviated this decrease. We also found protective effects of chlorogenic acid in neurons exposed to glutamate. Glutamate decreased the expression of PP2A subunit B and chlorogenic acid mitigated this decrease. Our results elucidated that chlorogenic acid performs neuroprotective functions and attenuates the reduction of PP2A subunit B by brain damage and glutamate-mediated excitotoxicity.

**Conclusions:**

We showed that chlorogenic acid attenuated the decrease of PP2A subunit B in ischemic injury and neurons exposed to glutamate. Since PP2A subunit B contributes to the protection of brain tissue, we can suggest that chlorogenic acid preserves neurons by modulating PP2A subunit B during ischemic damage.

**Supplementary Information:**

The online version contains supplementary material available at 10.1186/s42826-024-00196-5.

## Background

Cerebral ischemia has been reported to be a cause of cognitive impairment and dementia [1]. It is caused by blockage of the cerebral artery that supplies blood to the brain [2]. The lack of blood supply leads to neurological defects and eventually neuronal cell death [3]. Cerebral ischemia accelerates the reactive oxygen species and induces oxidative stress, causing damage to lipids and proteins, resulting in cell damage [4]. In addition, it leads to activation of inflammatory and apoptotic proteins that cause nerve damage, neuro-inflammation, and neuronal cell death [5].

Chlorogenic acid is a polyphenolic substance exists in citrus fruits, apples, and cocoa. Green roasted coffee beans have the highest concentration of chlorogenic acid compared to other natural sources [6]. Chlorogenic acid has health effects including anti-oxidative, anti-diabetic, and anti-cancer effects [7, 8]. It reduces cerebral infarction volume, brain edema, and neurological deficits and exerts neuroprotective effects in cerebral ischemia [9]. This substance reduces oxidative stress and improves neurobehavioral disorders during cerebral ischemic injury [10].

Protein phosphatase 2A (PP2A) is an important serine/threonine phosphatase that modulates the cell cycle and mobility, and signal pathways [11]. PP2A also contributes to gene transcription and metabolic functions. PP2A has three subunits, subunits A and C form dimers to bind to the regulatory B subunit [12]. The PP2A heterotrimeric protein phosphatase is ubiquitously expressed in mammalian cells. Among PP2A subunits, subunit B is mainly present in spinal cord and brain tissue. It regulates neurogenesis, and its overexpression play a protective role in ischemia–reperfusion injury [13, 14]. We identified a decrease in expression of PP2A subunit B in brain damage [15]. We hypothesized that chlorogenic acid exerts neuroprotective functions by modulating the expression of PP2A subunit B in stroke animal model and glutamate mediated toxicity. Thus, we investigated expression of PP2A subunit B by chlorogenic acid in stroke models and glutamate exposure neurons.

## Methods

### Animals grouping and drug treatment

Animals (220–230 g, 7 weeks, Sprague Dawley rats) were housed under controlled environmental conditions (25 °C, 12 h/12 h light cycle) for one week. They were divided into four groups: sham animals with PBS, sham animals with chlorogenic acid, MCAO animals with PBS, and MCAO animals with chlorogenic acid. PBS was used as a solvent, chlorogenic acid (30 mg/ kg) or PBS was intraperitoneally injected 2 h before the MCAO surgery [9]. The use of laboratory animals was performed with the approval of the University (GNU-22022-R0021).

### Middle cerebral artery occlusion (MCAO)

MCAO was operated as previously reported manuals [16]. Animals were anesthetized with Zoletil (50 mg/kg, Virbac, Carros, France) and carefully placed on the operating table in supine position. A midline of neck was cut to surgical operation. The common carotid artery (CCA), the external carotid artery (ECA), and internal carotid artery (ICA) were exposed and separated from nearby tissues and nerves. The superior thyroid artery (ST) and occipital artery (OA) were separated from adjacent tissues. Both ends of the ST and OA were ligated and a cut was given in the middle to block the supply of blood flow. Blood supply through the CCA was temporarily blocked with a microvascular clamp and the proximal end of ECA was severed. A nylon suture (4/0) with a round tip by heating was inserted in to the proximal end of the ECA and pushed forward until it reached the origin of middle cerebral artery. Inserted filament and the end of ECA were fixed with a black silk suture. The skin incision in the neck was closed with a black silk suture and animals were carefully placed in a separate cage. After 24 h, rats were sacrificed and the right cerebral cortex tissues were collected for further experiments.

### Two-dimensional gel electrophoresis and Western blot analyses

Tissues from the right cerebral cortex were lysed in lysis buffer (8 M urea, 4% CHAPS, 0.2% Bio-Lyte, 40 mM Tris–HCl) and centrifuged at 15,000 × g for 30 min at 4 °C. The supernatant was separated and precipitated in trichloroacetic acid at room temperature for 30 min. The obtained mixture was centrifuged and the supernatant was collected. Protein concentration was measured using a Bradford protein assay kit (Bio-Rad, Hercules, CA, USA). Protein (50 µg) per each group was used for proteomic study and protein (30 µg) per each group was used for Western blot analysis. Two-dimensional gel electrophoresis was performed according to previously described method [17]. Immunoblotting analysis was performed as follows. Sample of protein was loaded into the wells of a 10% sodium dodecyl sulfate–polyacrylamide gel and electrophoresis was performed. Proetins were tranfered to polyvinylidene difluoride membranes through semi-dry method and memebranes were blocked in a 5% skim milk solution for 1 h, washed with tris-buffered saline containing 0.1% tween-20 (TBST), and incubated with primary antibodies: anti-PP2A (1:1,000, Cell Signaling Technology, Danvers, MA, USA) and β-actin (1:1,000, Santa Cruz Biotechnology, Santa Cruz, CA, USA) overnight 4 °C. Membranes were incubated with horse radish peroxidase conjugated anti-mouse IgG or anti-rabbit IgG (1:5,000, Cell Signaling Technology) for 2 h and washed with TBST. They were reacted with chemiluminescence detection reagents (GE Healthcare, Little Chalfont, Buckinghamshire, UK) for protein bands detection. Band intensity was analyzed with Image J 1.50i (Media Cybernetics). Results were expressed as the relative integrated density of proteins to that of β-actin.

### Reverse transcription-polymerase chain reaction

Extracted total RNA (1 µg) was used to manufacture single-strand complementary DNA using the Superscript III first-strand system (Invitrogen, Carlsbad, CA, USA). Specific primers were used as follows: PP2A subunit B, F: 5'-CCTGGTATGCCAAACTCGAT-3'; R: 5'-ACAATAGCCACCTGGTCGTC-3' (product size, 223 bp), β-actin, F: 5'-TACAACCTTCTTGCAGCTCCTC-3'; R: 5'-CCTTCTGACCCATACCCACC-3' (product size, 205 bp). Amplification of the genes was completed in the following steps: denaturation of DNA at 94 °C for 5 min, denaturation at 94 °C for 30 s, annealing at 54 °C for 30 s, elongation at 72 °C for 1 min, and extension at 72 °C for 10 min. PCR reaction was performed for 30 cycles. The obtained PCR product was electrophorized in 1% agarose gel for 15 min using the Mupid-2 plus system (OPTIMA Inc. Tokyo Japan). Gels were obtained and images were quantified with Image J (Media Cybernetics, Bethesda, MD, USA).

### Immunohistochemical staining

Brain tissues were fixed in 4% neutral buffered paraformaldehyde solution, washed with tap water, dehydrated in graded ethyl alcohol series (70–100%), and cleaned in xylene. Brain slices were embedded in paraffin embedding center (Leica, Wetzlar, Germany) for 1 h. Paraffin blocks were cut into 4 µm thick using a rotary microtome (Leica, Wetzlar), and paraffin ribbons were mounted into slide glass. After the deparaffin process, the slides were microwaved for 2 min in a 10 mM sodium citrate buffer (pH 6.0) incubated with a 1% hydrogen peroxide solution prepared in methanol. The sections were reacted for 1 h with a 5% normal goat serum and incubated with anti-PP2A subunit B antibody (1:100, Cell Signaling Technology) at 4 °C. They were rinsed with PBS and incubated with a biotinylated goat anti-rabbit IgG (1:200 in PBS) for 2 h. The sections were incubated with avidin–biotin-peroxidase complex for 1 h through a Vector ABC Elite kit (Vector Laboratories Inc., Burlingame, CA, USA). They were washed three times with PBS and reacted with 3, 3′-diaminobenzidine tetrahydrochloride (DAB, Sigma-Aldrich, St. Louis, MO, USA). Hydrogen peroxide was added into DAB solution and staining was performed until the tissue color changed to brown. The slides were stained with hematoxylin solution (Sigma-Aldrich) The positive signals were counted under microscope (Olympus, Tokyo, Japan) The level of PP2A subunit B expression was expressed as a percentage of the number of PP2A subunit B positive cells to the number of total cells.

### Hippocampal cell line culture

HT-22 cells was grown with Dulbecco's Modified Eagle's Medium (DMEM, Gibco BRL, Gaithersburg, MD, USA). The cells were seeded with about 2 × 10^6^ cells in a 100 mm petri dish, and were cultured in a humidification incubator maintained at 37 °C and 5% CO_2_. When the confluency reached 70%, cells were exposed to glutamate (5 mM) and/or chlorogenic acid (Sigma-Aldrich, 10, 30, and 50 μM). Chlorogenic acid was treated 1 h prior to glutamate treatment. After 24 h of treatment, the cells were collected.

### Cell viability assay

The cells were seed on a 96-well plate (5 × 10^4^ cells per well) and cultured in a CO_2_ incubator for 24 h. Glutamate and/or chlorogenic acid treatment was performed as described above. The 3-(4,5-dimethylthiazol-2-yl)-2,5-diphenyltetrazolium bromide (MTT) was added to the cells then incubated in incubator at 37 °C for 4 h. After removing the MTT solution, 300 μl of dimethyl sulfoxide was added to each well. The optical density was examined at a wavelength of 570 nm. The values representing viable cells were expressed as a percentage of absorbance, the PBS group value was considered 100%.

### Statistical analysis

IBM SPSS statistical program (Chicago, IL, USA) was used for data analysis. Our data is presented as mean ± standard error of means (S.E.M.). The changes among the groups are measured through a two-way analysis of variance (ANOVA) followed by *post-hoc* Scheffe’s test. A *p*-value of less than 0.05 has been considered to be statistically significant.

## Results

### Modulation of PP2A subunit B by chlorogenic acid in middle cerebral artery occlusion (MCAO) damage

**Fig. 1:**
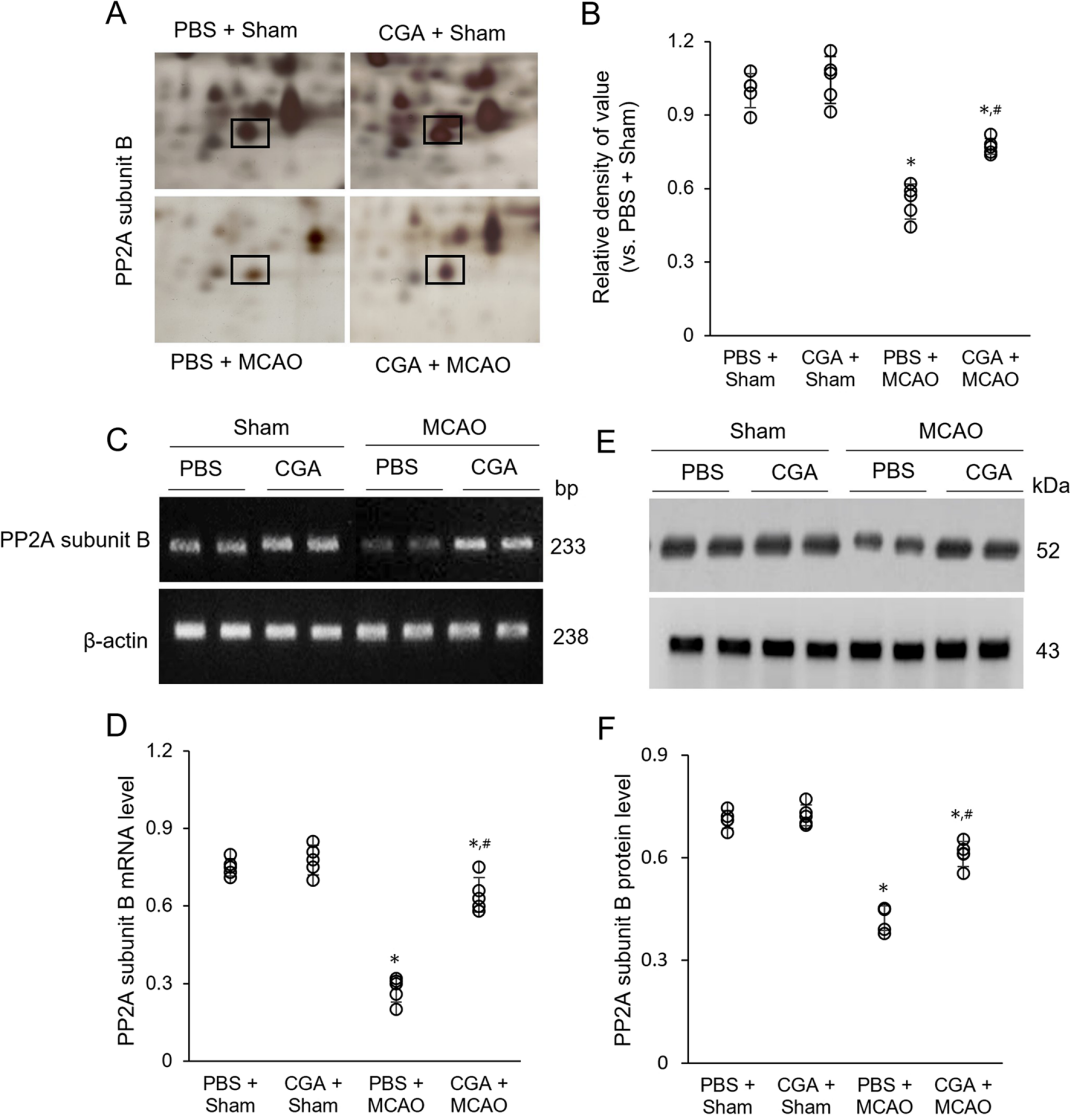
Chlorogenic acid mitigates the decrease of protein phosphatase 2A (PP2A) subunit B due to MCAO damage. Proteomic analysis (**A** and **B**), reverse transcription-PCR analysis (**C** and **D**), and Western blot analysis (**E** and **F**) of PP2A subunit B in the cerebral cortex of sham animals with phosphate buffer saline (PBS), sham animals with chlorogenic acid (CGA), middle cerebral artery occlusion (MCAO) animals with PBS, and MCAO animals with CGA. The square represents the PP2A subunit B protein spot. The intensity of the protein spot is given as the ratio of the intensity of each spot to the intensity of the PBS + sham animal (**B**). Mw and pI represent molecular weights and isoelectric points, respectively. The intensity analysis is indicated as a ratio of each subunit intensity to β-actin intensity (**D** and **F**). The data (*n* = 5 per group) are represented as the mean ± S.E.M. *p < 0.01 versus PBS + sham animals, #*p* < 0.01 versus PBS + MCAO animals

We showed the neuroprotective effects of chlorogenic acid before conducting this study. Chlorogenic acid significantly alleviated neurobehavioral disorders caused by middle cerebral artery occlusion (MCAO) damage (Additional file 1). In this study, we found that the expression of PP2A subunit B decreased in the cerebral cortex of MCAO animals was detected (Fig. 1A). The results of the proteomic study showed that the spot strength of PP2A subunit B was significantly decreased in MCAO animals, but this decrease was alleviated in chlorogenic acid-treated animals. The intensity of the PP2A subunit B spot in MCAO animals was 0.55 ± 0.04 and 0.77 ± 0.02 in phosphate buffer saline (PBS)- and chlorogenic acid-treated animals, respectively (F_3,16_ = 69.968, *P* < 0.01, Fig. 1B). There was no change in spot intensity in sham animals regardless of PBS and chlorogenic acid treatment. The PP2A subunit B expression was reduced in MCAO animals with PBS, but chlorogenic acid treatment mitigated this decrease (Fig. 1C). The mRNA levels of PP2A subunit B in MCAO animals were 0.28 ± 0.02 and 0.64 ± 0.03 in PBS- and chlorogenic acid-treated animals (F_3,16_ = 50.685, *P* < 0.01, Fig. 1D). The expression of these mRNAs were similar in sham animals. Western blot analysis also showed a decrease in PP2A subunit B expression in MCAO animals. Chlorogenic acid treatment mitigated this change caused by MCAO (Fig. 1E). The protein level of subunit B in MCAO animals was 0.42 ± 0.02 and 0.61 ± 0.02 in PBS- and chlorogenic acid-treated animals (F_3,16_ = 36.309, *P* < 0.01, Fig. 1F).

### Regulation of PP2A subunit B-positive cells by chlorogenic acid in MCAO damage

**Fig. 2:**
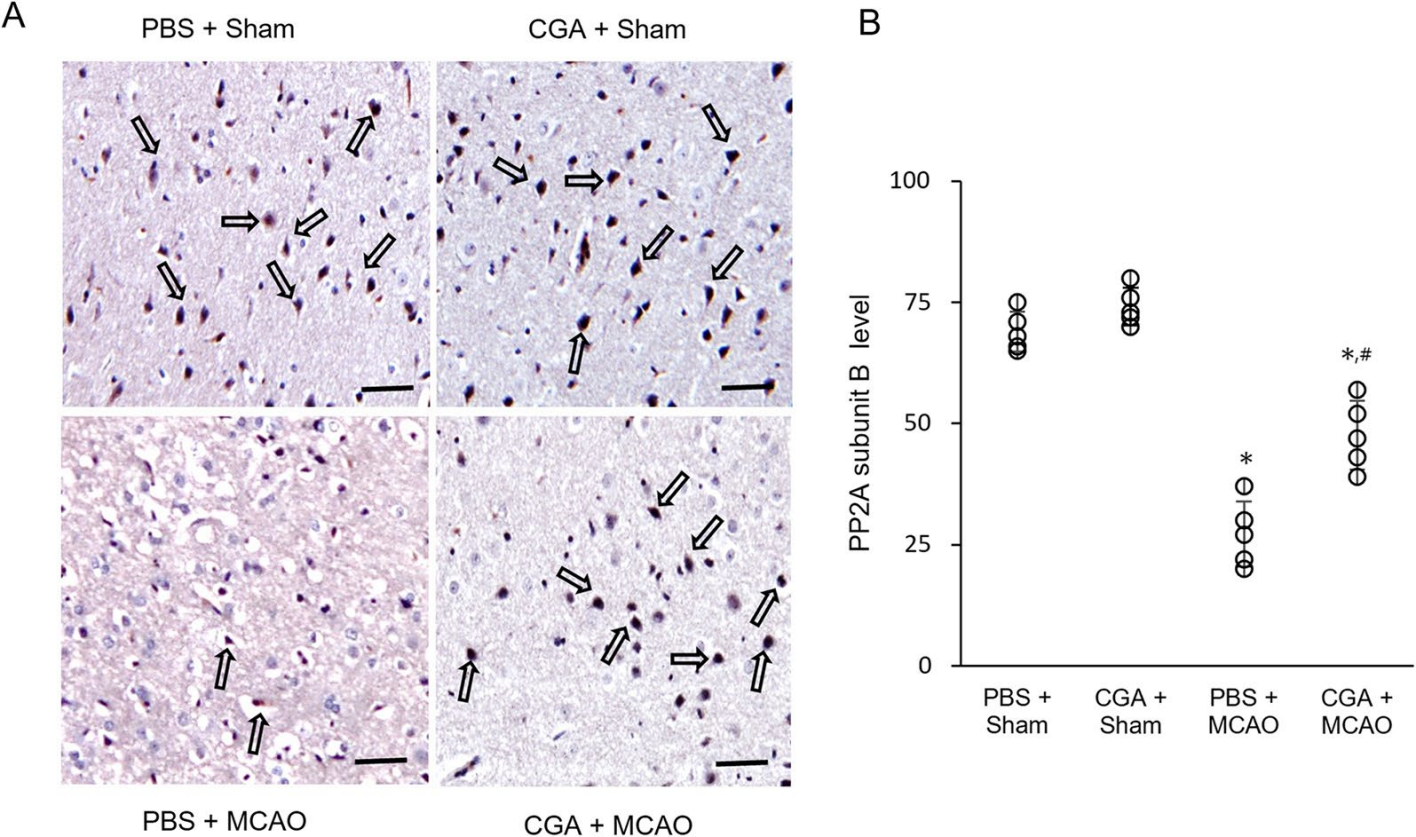
Chlorogenic acid migrates the reduction of protein phosphatase 2A (PP2A) subunit B protein expression due to MCAO damage. Representative images for immunohistochemical staining of PP2A subunit B protein in the right cerebral cortex of sham animals with phosphate buffer saline (PBS), sham animals with chlorogenic acid (CGA), middle cerebral artery occlusion (MCAO) animals with PBS, and MCAO animals with CGA (**A**). Open arrows explain the positive cells of PP2A subunit B. The number of PP2A subunit B-positive cells is expressed as a percentage of the number of positive cells relative to the total number of neurons (**B**). Scale bar: 100 μm. Data (*n* = 5 per group) are represented as the mean ± S.E.M. *p < 0.01 versus PBS + sham animals, #*p* < 0.01 versus PBS + MCAO animals

Immunohistochemical staining showed that the number of PP2A subunit B-positive cells was decreased in MCAO animals. Chlorogenic acid treatment alleviated the PP2A subunit B decrease caused by MCAO (Fig. 2A). The number of positive cells in MCAO animals was 27.67 ± 4.98 and 47.66 ± 3.84 in PBS- and chlorogenic acid-treated animals, respectively (F_5,24_ = 8.570, *P* < 0.01, Fig. 2B). The number of positive cells was similar in sham animals.

### Regulation of PP2A subunit B protein by chlorogenic acid in glutamate-exposed neurons

**Fig. 3:**
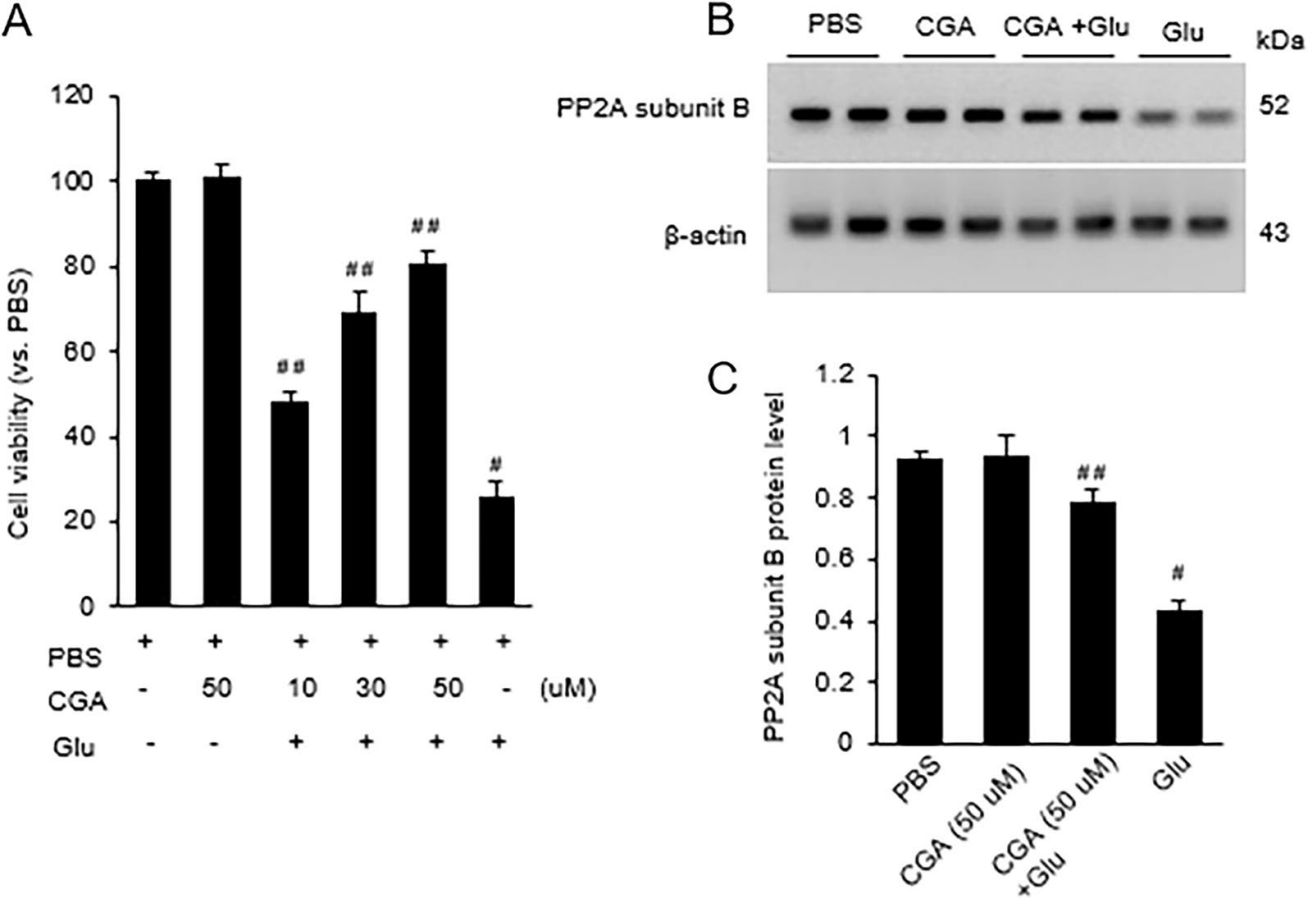
Chlorogenic acid mitigates the decrease of cell viability and protein phosphatase 2A (PP2A) subunit B expression due to glutamate exposure. Cell viability (**A**) and Western blot (**B** and **C**) analyses of PP2A subunit B in HT22 cells with glutamate (Glu) and/or chlorogenic acid (CGA). Cell viability was represented as a percentage of the PBS set at 100 (**A**). The levels of protein is expressed as the intensity of PP2A subunit B to intensity of actin (**C**). Data (*n* = 5) are represented as mean ± S.E.M. *p < 0.01 versus PBS. #*p* < 0.01 versus Glu

In culture system of hippocampal neuronal cell line (HT-22 cells), we showed a decrease in cell viability after exposure to glutamate. However, chlorogenic acid treatment attenuated the decrease in cell viability due to glutamate toxicity. The alleviative effect of chlorogenic acid was dose-dependent. The cell viability of the PBS-treated group was set to 100. Cell viabilities were 101.3 ± 3.1 in the chlorogenic acid-only group and 48.1 ± 2.4, 69.3 ± 4.8, and 80.6 ± 3.2 in glutamate group with 10, 30, and 50 µM of chlorogenic acid, respectively. Cell viability was 25.8 ± 3.7 in the glutamate-treated group (F_3,16_ = 15.848, *P* < 0.01, Fig. 3A). Western blot results showed that glutamate decreased the expression of PP2A subunit B, and chlorogenic acid treatment alleviated this decrease (Fig. 3B). PP2A subunit B protein level was 0.43 ± 0.03 in the glutamate only group and 0.78 ± 0.05 in the glutamate and chlorogenic acid (50 µM) co-treatment group (F_3,16_ = 35.749, *P* < 0.01, Fig. 3C).

## Discussion

We reported that chlorogenic acid performs as protective function in stroke animal model [10]. Chlorogenic acid treatment mitigated MCAO-induced neurological behavioral disorders, the increase in oxidative stress generation, infarction, edema, and histopathological changes [10]. Because our previous studies have already reported the protective effect of chlorogenic acid through various experimental techniques. We only provided supplement data to explain this information. We previously showed a decrease in PP2A subunit B expression in the MCAO stroke animal model [15]. Additionally, we explained the mitigation of PP2A subunit B reduction in chlorogenic acid. We focused on the importance of subunit B and continued to investigate the changes in expression of the subunit B protein in the presence of chlorogenic acid in ischemic injury. Chlorogenic acid mitigates MCAO-induced reduction of PP2A subunit B protein expression. These results provide evidence that cerebral ischemia significantly reduced PP2A subunit B mRNA and protein expression in the ischemic cerebral cortex, and that chlorogenic acid treatment mitigated the decrease of PP2A subunit B induced by MCAO.

Glutamate is an important neurotransmitter and performs a crucial role in metabolism [18]. It also acts as an important factor in neuronal differentiation, synapse conservation, and plasticity [18]. However, excessive glutamate exposure increases intracellular calcium concentration and disrupts cell structure, resulting in cell death [19]. Since brain is a sensitive organ that consumes oxygen and glucose restrictively, a decrease of blood flow causes energy depletion in nerve cells. ATP deficiency causes ion gradient imbalance and interferes with glutamate reuptake and secretion. As a result, glutamate accumulates in the synapse, overexcites NMDA receptor of the postsynaptic neurons, and results in cell death. Furthermore, activation of NMDA receptor induces an increase of calcium in the postsynaptic neuron and accelerates apoptosis [20]. MCAO was used to establish an animal model of ischemic stroke. Glutamate treatment was used to induce nerve cell damage similar to in vivo experiments, and to cause neurotoxicity of nerve cells in vitro study. In this study, we showed that glutamate treatment reduced cell viability, whereas chlorogenic acid treatment alleviated the reduction of cell viability by glutamate toxicity in a dose-dependent manner. In addition, glutamate treatment reduced the expression of PP2A subunit B, and chlorogenic acid treatment attenuated this reduction. A reduction in PP2A subunit B induces apoptosis and cell damage. This reduction in PP2A subunit B can be caused by damage mechanisms such as excessive ROS, and this decrease in PP2A can cause excitatory neurotoxicity by regulating the glutamatergic system [21]. Moreover, the overexpression of PP2A subunit B56α exerts a protective effect against ischemic and reperfusion injury [14]. This subunit regulates the phosphorylation of calcium regulatory proteins, activation of protective signaling programs, and resistance to ischemic stress [14]. This study showed that chlorogenic acid treatment mitigates the reduction of PP2A subunit B by glutamate toxicity.

Chlorogenic acid reduces the increase in inflammatory and apoptotic proteins induced by cerebral ischemia [10, 22]. We have previously provided evidence for various beneficial effects of chlorogenic acid such as anti-oxidation, anti-inflammation, and apoptosis in cerebral ischemia via activation of the survival pathway [12, 22]. This study showed that chlorogenic acid significantly attenuated ischemia-induced reduction of this protein expression. However, we did not show the regulatory mechanism of this protein by chlorogenic acid treatment. We think the further experiment is required to explain the mechanism. We previously showed that chlorogenic acid performs a neuroprotective role in cerebral ischemia by modulating various proteins [17]. However, in sham animals, chlorogenic acid-treated animals did not show significant changes compared to PBS-treated animals. These findings showed that chlorogenic acid has a minimal off-target effect at the protein expression level. The maintenance of PP2A subunit B expression is critical for cell survival. Thus, our findings can demonstrate that chlorogenic acid mitigates the modulation of PP2A subunit B during MCAO damage and glutamate treatment, and that conservation of PP2A subunit B perform to the protective effect of chlorogenic acid.

## Conclusions

Chlorogenic acid act as an important agent to perform the neuroprotective function in brain damage and glutamate toxicity by regulation of PP2A subunit B. We elucidated that the expression of PP2A subunit B is involved in the protective mechanism of chlorogenic acid.

### Supplementary Information

**Additional file 1**. Chlorogenic acid alleviates neurobehavioral disorder due to MCAO damage. Neurobehavioral scores test (A) and adhesive removal test (B) in the sham animals with phosphate buffer saline (PBS), sham animals with chlorogenic acid (CGA), middle cerebral artery occlusion (MCAO) animals with PBS, and MCAO animals with CGA (A). Neurological deficit scores were assessed according to the following postures: normal posture (0), no extension of the contralateral forelimb (1), frequently rotation to the contralateral direction (2), not moving or trying to move the contralateral side of body (3), no conscious movements (4). Adhesive stickers were attached to both the forelimbs of animals and the time taken for the animals to remove each adhesive sticker was recorded. CGA mitigated the neurobehavioral disorder caused by MCAO damage. Data (*n* = 20 per group) are represented as mean ± S.E.M. *p < 0.01 vs. PBS + sham animals, #p < 0.01 vs. PBS + MCAO animals.

## Data Availability

All data are given in the current manuscript.

## Abbreviations

CCA: Common carotid artery
CGA: Chlorogenic acid
CHAPS: 3-[(3-Cholamidopropyl)dimethylammonio]-1-propanesulfonate.
DTT: Dichlorodiphenyltrichloroethane
ECA: External carotid artery
ICA: Internal carotid artery
IPG: Immobilized pH-gradient
MCAO: Middle cerebral artery occlusion
MALDI-TOF: Matrix-assisted laser desorption/ionization-time of flight
PBS: Phosphate-buffered saline
PCR: Polymerase chain reaction
PP2A: Protein phosphatase 2A
OA: Occipital artery
ROS: Reactive oxygen species
SDS: Sodium dodecyl sulphate
ST: Thyroid artery
TBST: Tris-buffered saline containing 0.1% tween-20
2-DE: Two-dimensional gel electrophoresis
DAB, Sigma-Aldrich: 3,3′-Diaminobenzidine tetrahydrochloride
