# Supplementary figures and images for: Chlorogenic acid regulates the expression of protein phosphatase 2A subunit B in the cerebral cortex of a rat stroke model and glutamate-exposed neurons
Source: Lab Anim Res. 2024 Mar 1;40:8. doi: 10.1186/s42826-024-00196-5 (PMC10905799; doi:10.1186/s42826-024-00196-5)

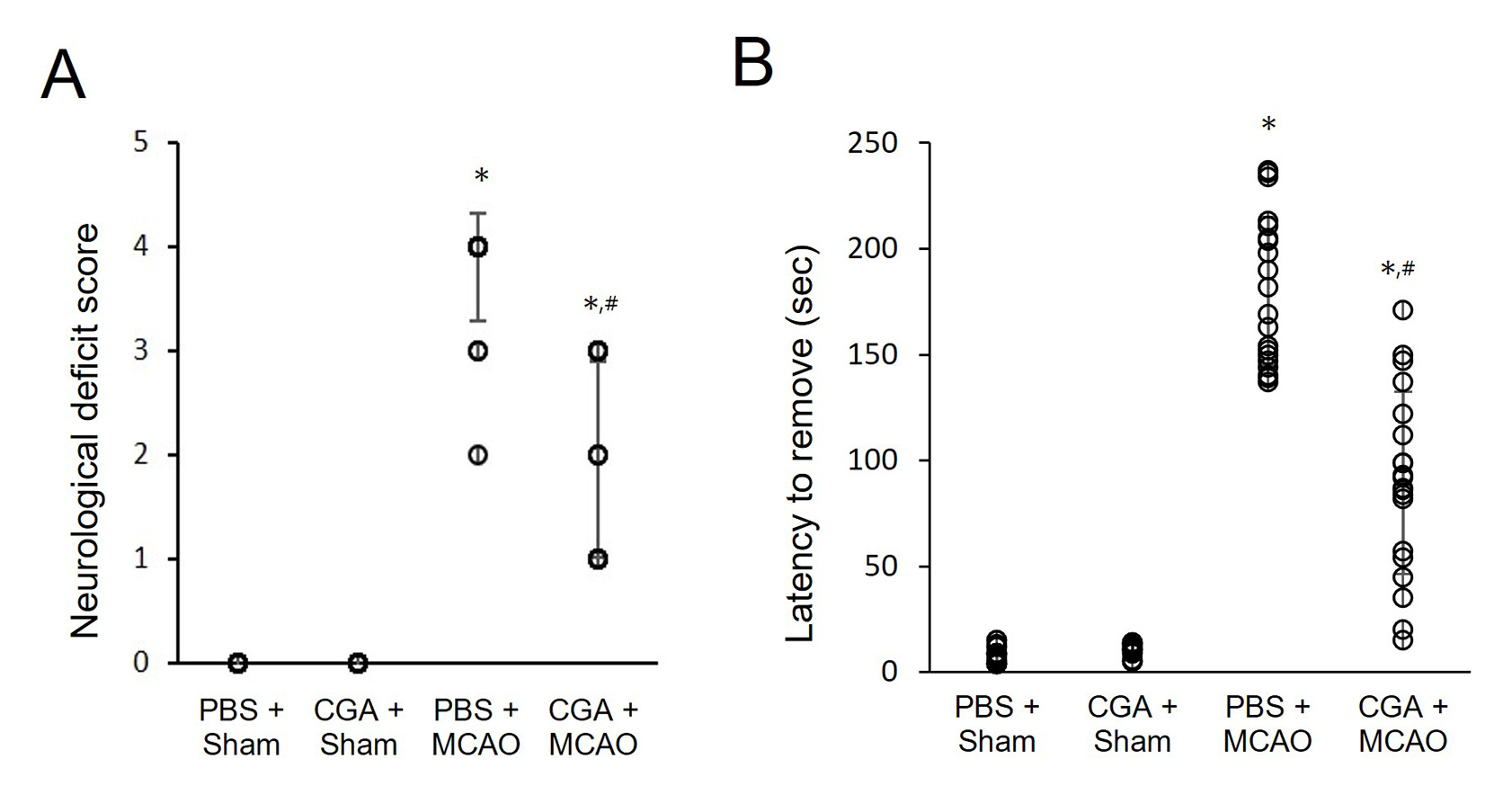

Supplement: Supplementary file 1 — Additional file 1. Chlorogenic acid alleviates neurobehavioral disorder due to MCAO damage. Neurobehavioral scores test (A) and adhesive removal test (B) in the sham animals with phosphate buffer saline (PBS), sham animals with chlorogenic acid (CGA), middle cerebral artery occlusion (MCAO) animals with PBS, and MCAO animals with CGA (A). Neurological deficit scores were assessed according to the following postures: normal posture (0), no extension of the contralateral forelimb (1), frequently rotation to the contralateral direction (2), not moving or trying to move the contralateral side of body (3), no conscious movements (4). Adhesive stickers were attached to both the forelimbs of animals and the time taken for the animals to remove each adhesive sticker was recorded. CGA mitigated the neurobehavioral disorder caused by MCAO damage. Data (n = 20 per group) are represented as mean ± S.E.M. *p < 0.01 vs. PBS + sham animals, #p < 0.01 vs. PBS + MCAO animals. [file 42826_2024_196_MOESM1_ESM.tif]
